# Hyperthermic Intraperitoneal Chemotherapy plus Intravenous Chemotherapy of Paclitaxel with or without Sintilimab in Gastric Cancer: A Comparative Study

**DOI:** 10.1155/2022/3054485

**Published:** 2022-02-22

**Authors:** Zao Zhang, Mei-Ying Ning, Dong Han, Li Li, Zhuang-Zhuang Li

**Affiliations:** Department of Pharmacy, Cangzhou City Center Hospital, Cangzhou, Hebei Province, China

## Abstract

**Objective:**

To compare the clinical efficacy of hyperthermic intraperitoneal chemotherapy (HIPEC) plus intravenous chemotherapy of paclitaxel with or without sintilimab in peritoneal metastasis of gastric cancer.

**Methods:**

A total of 120 patients assessed for eligibility with peritoneal metastasis of gastric cancer treated in the oncology department of our hospital from January 2019 to June 2020 were recruited. They were concurrently randomly assigned in a 1 : 1 ratio to receive HIPEC plus sintilimab-paclitaxel intravenous chemotherapy (study group) or plus paclitaxel intravenous chemotherapy only (control group).

**Results:**

The objective remission rate (ORR) of ascites in the study group was significantly higher than that in the control group. Subgroup analysis showed that an age ≤60 years or well-differentiated tumors were associated with better objective remission. After treatment, significantly higher Karnofsky Performance Status (KPS) scores were observed in the study group versus those of the control group. Adverse events reported were comparable between groups. The study group obtained longer 12-month progression-free survival (PFS) and overall survival (OS) than those of the control group.

**Conclusion:**

On top of HIPEC, intravenous chemotherapy with sintilimab and paclitaxel constitute an effective alternative for patients with peritoneal metastasis of gastric cancer to enhance ascites remission, ameliorate the quality of life, and prolong survival, versus with paclitaxel alone.

## 1. Introduction

Gastric cancer is the second leading cause of cancer-related deaths. More than 90% of gastric cancers are gastric adenocarcinomas, with atypical early symptoms, and often progress to the middle and advanced stages at the time of diagnosis, and the 5-year survival rate is only 20% [1]. Gastric cancer with peritoneal metastasis refers to the invasion of gastric cancer cells to the serosal layer. Surgical resection is the mainstay of treatment for early gastric cancer but benefits little in the advanced stage. Research has shown that peritoneal metastasis is associated with about 14% of newly diagnosed gastric cancer patients and up to 39–43% of advanced cases [2]. Moreover, the poor prognosis of peritoneal metastasis, mirrored by concomitant malignant ascites and short median survival of 4 to 6 months, compromises the quality of life and survival of patients [3]. Peritoneal metastasis is usually treated with systemic chemotherapy, in which platinum and fluoropyrimidine are the first-line drugs, and paclitaxel plus ramucirumab are the second-line ones [4]. However, in light of a low drug concentration in the peritoneum, hyperthermic intraperitoneal chemotherapy (HIPEC) may enhance the treatment efficiency.

HIPEC, with the merits of the pharmacokinetics of the peritoneal-plasma barrier, allows sufficient contact of the cytotoxic drugs with tumor tissues and cells, thereby effectively killing cancer cells and achieving therapeutic effects [5]. Paclitaxel maintains a high drug concentration in a long duration in HIPEC thanks to its unique pharmacological properties and inhibits abdominal metastasis of various cancers [6]. In addition, its strong antiproliferative activity prevents chemical adhesions in the peritoneal cavity and allows reiterative HIPEC. Hyperthermic intraperitoneal chemotherapy (HIPEC) with paclitaxel has obtained a desirable outcome in peritoneal metastasis of ovarian cancer and thus was considered a first-line regimen. A meta-analysis confirmed the survival benefits of HIPEC [7], whereas inconsistent findings also exist [8, 9]. Sintilimab is an immune checkpoint inhibitor that binds to programmed death-1 (PD-1), blocks the binding to the receptor, and restores the body's normal antitumor function, thereby achieving tumor control and tumor cells scavenging [10]. Sintilimab has been widely used in patients with refractory Hodgkin's lymphoma, and its application in other solid tumors also receives extensive attention [11]. This study was to investigate the safety and effectiveness of sintilimab plus HIPEC in patients with peritoneal metastasis of gastric cancer.

## 2. Materials and Methods

### 2.1. General Information

A total of 120 eligible patients with peritoneal metastasis of gastric cancer treated in the oncology department of our institution from January 2019 to June 2020 were recruited and assigned to either a study group (*n* = 60) or a control group (*n* = 60). The study was approved by the hospital ethics committee, with the approved no. of 2018-LCC293/45 and was conducted as per the ethical guidelines of the *Declaration of Helsinki* [12]. All subjects provided a signed informed consent form before randomization.

### 2.2. Inclusion and Exclusion Criteria

#### 2.2.1. Inclusion Criteria

(1) Patients aged 18 to 75 years; (2) patients with gastric cancer or gastroesophageal junction cancer confirmed by histopathology; (3) patients with a diagnosis of peritoneal metastasis or peritoneal lavage confirmed by laparoscopy; (4) patients with an estimated survival time of ≥6 months; and (5) patients with Eastern Cooperative Oncology Group (ECOG) score 0∼1 point.

#### 2.2.2. Exclusion Criteria

(1) Patients with other distant metastases other than peritoneal metastasis; (2) pregnant or lactating women; (3) patients with other uncontrolled malignant tumors in the past 5 years before randomization; (4) patients with other serious or uncontrolled tumors; and (5) patients with allergies to the drugs used in this study.

### 2.3. Research Design

All patients underwent laparoscopic palliative resection for gastric cancer. After the operation, drainage tubes with multiple side holes were indwelt on the diaphragmatic surface of the liver, splenic fossa, and pelvis, secured on the left upper abdomen, left lower wall, and right upper abdomen. HIPEC was performed 48 hours postoperatively, for 4 times in total on alternate days. Paclitaxel (Hainan Sinochem United Pharmaceutical Industry Co., Ltd.; approval no. H20057065) 175 mg/m^2^, divided into 3 total doses, was dissolved in 3000 ml of 0.9% sodium chloride solution. During HIPEC, the circulation pipeline was connected to the 4 HIPEC-dedicated catheters inserted during the operation in a two-in and two-out manner, which allowed the thermal perfusion fluid to be instilled into the peritoneal cavity, with the perfusion speed of 500∼600 ml/min, the perfusion temperature of (43 ± 0.1)°C, and the perfusion duration of 60 min. The control group was treated with intravenous injection of paclitaxel at a dose of 40 mg/m^2^ on day 1 and day 8 after surgery. Cisplatin (40 mg/m^2^) was injected intravenously on day 1 postoperatively, and tegafur, gimeracil, and oteracil potassium capsules (Shandong New Times Pharmaceutical Co., Ltd., approval no. H20080802) 50 mg/m^2^ were given orally every day from day 1 to day 14. On the treatment basis of the control group, the study group was additionally given sintilimab (Xinda Biopharmaceutical Co., Ltd., approval no. CXSS2100007): sintilizumab 200 mg was intravenously administered, every 3 weeks as maintenance treatment. The treatment continued until documented disease progression, withdrawal of informed consent, or study end. All patients were treated continuously for 12 weeks. Allergies and vomiting were routinely prevented before chemotherapy, and the peritoneal cavity was hydrated for 3 days after chemotherapy.

### 2.4. Primary Outcome

#### 2.4.1. Objective Curative Effect Evaluation

Before and after treatment, the objective curative effect was evaluated per the response evaluation criteria in solid tumors guidelines version 1.1 (RECIST 1.1) [13] and divided into complete remission (CR), partial remission (PR), stable disease (SD), and progressive disease (PD). CR: the ascites completely disappear and last for more than 4 weeks; PR: the ascites reduce by more than 50% for 4 weeks; SD: the ascites reduce by less than 50% or the increase by no more than 25%; PD: the ascites increase by more than 25% compared to that before treatment. Objective remission (OR) = CR + PR.

#### 2.4.2. Long-Term Curative Effect

Patients were followed up monthly for 12 months. Progression-free survival (PFS) and overall survival (OS) were used to evaluate the long-term curative effect of patients. PFS is defined as no new distant metastases; OS is the total survival time of the patient.

### 2.5. Secondary Outcome

#### 2.5.1. Quality of Life

Karnofsky Performance Status (KPS) [14] was used to evaluate the functional status of patients after treatment. The total score is 100 points, and a higher score indicates better functional status. A score of 0 points is interpreted as death, 10 points as life-threatening, 20 points as a serious condition that requires hospitalization, 30 points as bed rest and hospitalization are needed but not life-threatening, 40 points as complete incapability of self-care, 50 points as requiring assistance and care in self-care, 60 points as being able to conduct most self-care, 70 points as no obstacles in self-care, yet inability to work, 80 self-care as being able to lead a basic normal work and life but easily tired, 90 self-care as no obstacles in leading a basic normal work and life, and 100 points as no symptoms.

#### 2.5.2. Adverse Reactions

Common Terminology Criteria Adverse Events (CTCAE 4.0) [15] were used to evaluate adverse events (AEs) during treatment which were classified into I ∼ V grades. Grade I is mild where it is an asymptomatic or mild symptom, only be detected by clinical or diagnostic findings, and no treatment is required. Grade II is moderate where shows indications of minimal, local, or noninvasive, and activities of daily living are restricted. Grade III is severe or of great medical significance where there are no immediate life-threatening conditions and shows indications of hospitalization or prolonged hospitalization, disability can be foreseen, and self-support activities of daily living are restricted. Grade IV is life-threatening and requires emergency treatment. Grade V is death.

### 2.6. Statistical Methods

The data in the present study were organized by using the Epidata software, analyzed using SPSS 22.0, and visualized into matching graphics by using GraphPad Prism 9.0. The measurement data were expressed as ($\bar{x}$ ± *s*); paired-samples *t*-test was adopted for the comparison of different timepoints in group and independent-samples *t*-test was adopted for the comparison of different groups. The count data were expressed as the rate and examined by the chi-square test. The survival curve was estimated using the Kaplan–Meier method, and the stratified Cox regression model was to estimate the hazard ratio (HR). The difference was assumed at *α* = 0.05.

## 3. Results

### 3.1. Comparison of General Data

The baseline data of the two groups of patients, such as age, gender, tumor pathological type, ECOG score, and degree of differentiation, were not statistically different (all *P* > 0.05) (Table 1).

### 3.2. Evaluation of Objective Curative Effect

**Figure 1:**
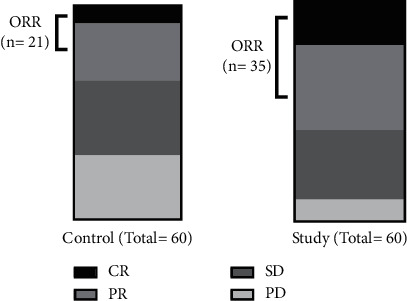
Comparison of the objective curative effect. CR, complete remission; PR, partial remission; SD, stable disease; PD, progressive disease; ORR, objective reaction rate.

The control group had 5 cases of CR, 16 cases of PR, 21 cases of SD, 18 cases of PD, and a total of 21 cases of OR, with an ORR of 35.00%. The study group had 12 cases of CR, 23 cases of PR, 19 cases of SD, and 6 cases of PD, and a total of 35 cases with OR, with an ORR of 58.33%. The results of chi-square analysis showed that the ORR of the study group was significantly higher than that of the control group (*χ*2 = 6.563, *P* = 0.010) (Figure 1).

### 3.3. Subgroup Analysis of ORR

Among all subjects, 56 patients obtained objective remission. It was found that an age ≤60 years or well-differentiated tumors were associated with better objective remission (*P* < 0.05), whereas there was no significant difference in ORR between different genders, pathological types, and ECOG score (all *P* > 0.05) (Table 2).

### 3.4. Comparison of the Quality of Life

The KPS scores did not statistically differ between the two groups before treatment (49.28 ± 8.28 vs 52.26 ± 9.59) (*P* > 0.05). After treatment, the KPS score of the control group was 62.25 ± 12.08 and of the study group was 67.16 ± 11.95 (all *P* < 0.05) (Table 3).

### 3.5. Comparison of the Long-Term Clinical Efficacy

The 6-month and 12-month PFS of the control group were 76.67% (46/60) and 26.67% (16/60), respectively, and the study group were 83.33% (50/60) and 45.00% (27/60); the 12-month PFS of the study group was significantly higher than that of the control group (*P* = 0.036). The 6-month and 12-month OS of the control group were 85.00% (51/60) and 56.67% (34/60), respectively, and the study group were 91.67% (55/60) and 80.00% (48/60), respectively. The 12-month OS of the study group was significantly higher than that of the control group (*P* = 0.006) (Table 4).

### 3.6. Comparison of PFS and OS

**Figure 2:**
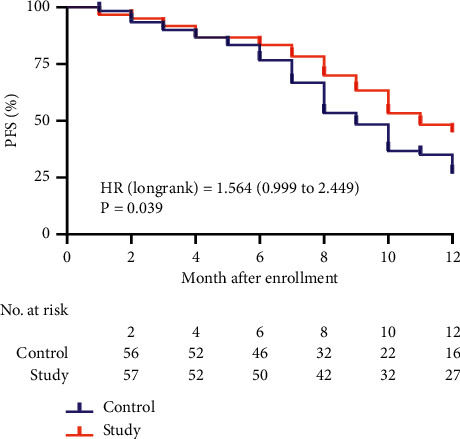
Kaplan–Meier survival curve of PFS. PFS, progression-free survival; HR, hazard ratio.

**Figure 3:**
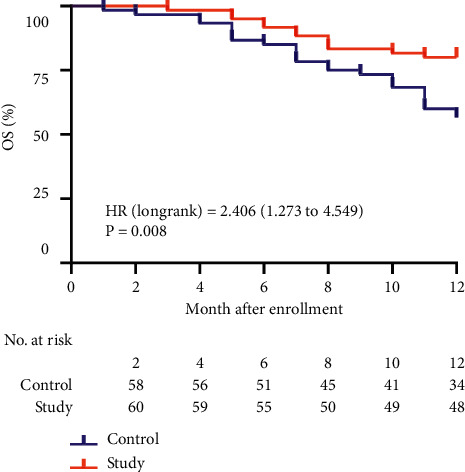
Kaplan–Meier survival curve of OS. OS, overall survival; HR, hazard ratio.

After 12 months of follow-up, the median PFS in the control group was 8 months, and the study group was 10 months (Figure 2). There was a significant difference between the two groups in PFS, HR = 1.564 (95% CI 0.999 to 2.449, *P* = 0.039). After 12 months of follow-up (Figure 3), there was a significant difference between the two groups in OS, HR = 2.406 (95% CI 1.273 to 4.549, *P* = 0.039).

### 3.7. Comparison of Adverse Reactions

As shown in Table 5, the control group had 53 cases of AEs, and 33 cases had ≥3 AEs; the study group had 51 cases of AEs, and 26 cases had ≥3 AEs. There was no significant difference in AEs between the two groups (all *P* > 0.05).

## 4. Discussion

Gastric cancer is a common malignancy and the second leading cause of cancer-related deaths worldwide. The peritoneum is the most common metastatic site after radical gastrectomy. Research has shown that the median survival time of patients with peritoneal metastasis is only 4 months and of those without peritoneal metastasis is 14 months [16]. Early diagnosis of peritoneal metastasis of gastric cancer remains a pressing issue to be addressed. Malignant ascites are the primary symptom of peritoneal metastasis. The 2-year survival of peritoneal metastases of gastric cancer is poor despite surgical resection with curative intent [2].

In the present study, patients in the control group received an intravenous injection of paclitaxel plus HIPEC, where an improved long-term efficacy of the patients with a 1-year survival rate of 56.67% was observed, confirming the robustness of HIPEC in treating peritoneal metastasis. The promising results can be attributed to the direct contact of HIPEC drugs with tumor cells to achieve cancer cells elimination and ascites control, and the favorable effectiveness and a safety profile of HIPEC due to the lower permeability of the peritoneum than the plasma clearance rate and higher concentration of HIPEC drugs than the peripheral blood. Furthermore, HIPEC drugs also enter the liver through the portal vein, resulting in a reduced risk of liver metastasis [17]. In this study, the use of paclitaxel as a HIPEC drug was mainly based on the following reasons. Paclitaxel, extracted from the plant *Taxus chinensis*, acts on the tubulin system and microtubules, inhibits the depolymerization of microtubules, promotes tubulin, and accelerates the apoptosis of cancer cells. It serves as a first-line chemotherapy drug for advanced gastric cancer. Moreover, owing to its large molecular weight and antiproliferative activity, paclitaxel can reduce chemical adhesion while maintaining a high drug concentration for a long time [18, 19].

To enhance the efficacy, the study group was additionally given an intravenous injection of sintilimab on top of HIPEC plus intravenous injection of paclitaxel, and the results reported a significantly higher ascites remission rate and higher long-term efficacy, with the 1-year survival rate reaching 80%. Sintilimab is a PD-1 inhibitor and an immune checkpoint inhibitor that blocks the interaction between PD-1 and its ligand PD-L1, to restore the body's normal antitumor immune response and achieve tumor control and elimination [10]. The efficiency of sintilimab is well-recognized in Hodgkin's lymphoma, and its application in other solid tumors also receives extensive attention [20]. The ORIENT-15 study reported an excellent outcome of sintilimab combined with chemotherapy concerning OS and PFS and safety profile in patients with esophagogastric cancer regardless of PD-L1 expression [21]. Similar to the present study, Jiang et al. [22] used sintilimab combined with oxaliplatin/capecitabine as the first-line treatment for locally advanced or metastatic gastroesophageal junction adenocarcinoma and reported an objective response rate of 85.0%, and a median PFS of 7.5 months, which potentiates the application effect of sintilimab in advanced gastric cancer. To our best understanding, the PD-L1 expression status is closely related to the efficacy of PD-1 inhibitors. However, this study failed to evaluate the PD-L1 expression, which requires further investigation.

To sum up, on top of HIPEC, intravenous chemotherapy of sintilimab and paclitaxel constitute an effective alternative for patients with peritoneal metastasis of gastric cancer to enhance ascites remission, ameliorate the quality of life, and prolong survival, versus with paclitaxel alone.

## Figures and Tables

**Table 1:** Comparison of the general data.

	Control (*n* = 60)		Study (*n* = 60)	
	No.	%	No.	%
*Age, years*				
≤60	26	43.33	19	31.67
>60	34	56.67	41	68.33

*Gender*				
Male	35	58.33	39	65.00
Female	25	41.67	21	35.00

*Pathological type*				
Adenocarcinoma	55	91.67	57	95.00
Others	5	8.33	3	5.00

*ECOG score*				
0	42	70.00	39	65.00
1	18	30.00	21	35.00

*Differentiations*				
Low	11	18.33	14	23.33
Medium/High	49	81.67	46	76.67

ECOG, Eastern Cooperative Oncology Group.

**Table 2:** Subgroup analysis of ORR.

	Total no.	No. of OR	*χ* ^2^	*P* value
*Age, years*			9.143	0.003
≤60	45	29		
>60	75	27		

*Gender*			0.333	0.564
Male	74	33		
Female	46	23		

*Pathological type*			0.959	0.327
Adenocarcinoma	112	50		
Others	8	6		

*ECOG score*			2.765	0.096
0	81	42		
1	39	14		

*Differentiations*			6.519	0.011
Low	25	6		
Medium/High	95	50		

ECOG, Eastern Cooperative Oncology Group; ORR, objective reaction rate.

**Table 3:** Comparison of the KPS scores.

	Before	After	*t*	*P* value
Control group (*n* = 60)	49.28 ± 8.28	62.25 ± 12.08	6.860	<0.001
Study group (*n* = 60)	52.26 ± 9.59	67.16 ± 11.95	7.533	<0.001
*t*	1.822	2.194		
*P* value	0.071	0.027		

KPS, Karnofsky Performance Status.

**Table 4:** Comparison of long-term outcome.

	PFS (%)		OS (%)	
	6 month	12 month	6 month	12 month
Control group (*n* = 60)	46	16	51	34
Study group (*n* = 60)	50	27	55	48
*χ* ^2^	1.768	4.385	1.294	7.548
*P* value	0.184	0.036	0.255	0.006

PFS, progression-free survival; OS, overall survival.

**Table 5:** Comparison of the adverse effects.

	All grade AEs		Grade ≥3 AEs	
	No.	%	No.	%
Control group (*n* = 60)	53	83.33	33	55.00
Study group (*n* = 60)	51	85.00	26	43.33
*χ* ^2^	0.289		1.634	
*P* value	0.296		0.101	

AEs, adverse events.

## Data Availability

The datasets used during the present study are available from the corresponding author upon reasonable request.
